# Language as a Window to the Mind: Parental Mental State Language in Relation to Deaf and Hard-of-Hearing Children’s Social–Emotional Skills

**DOI:** 10.3390/bs15111558

**Published:** 2025-11-14

**Authors:** Lizet Ketelaar, Nadine P. W. D. de Rue, Eva de Boer, Evelien Dirks

**Affiliations:** 1 Dutch Foundation for the Deaf and Hard of Hearing Child, 1073 GX Amsterdam, The Netherlands; edeboer@nsdsk.nl (E.d.B.); edirks@nsdsk.nl (E.D.); 2 Royal Dutch Kentalis, 5271 SW Sint-Michielsgestel, The Netherlands; n.derue@kentalis.nl; 3 Department Tranzo, Tilburg University, 5037 AB Tilburg, The Netherlands

**Keywords:** theory of mind, emotion understanding, social cognition, deafness, hearing loss

## Abstract

Early parent–child interactions are crucial for children’s social–emotional development. Mental state talk (MST)—language referring to thoughts, feelings, and intentions—is a key contributor. MST may be reduced in hearing parents of deaf or hard-of-hearing (DHH) children, who face unique communication challenges. Yet, existing research on MST in hearing parents of DHH children and on MST use by DHH children themselves is limited and fragmented, often focusing on MST quantity in a single context. Few studies have examined MST quality, variation across contexts, or its relationship with children’s social–emotional functioning. This study addresses these gaps by investigating MST quantity and quality across two activities and its associations with children’s MST and social–emotional development. Forty-eight hearing parent–DHH child dyads (ages 2–5) participated. MST was studied during storybook reading and free play. Children completed tasks on emotion understanding and theory of mind; parents reported on MS vocabulary and family characteristics. The results showed that parents adjusted MST complexity based on children’s age but not audiological characteristics. MST varied by activity, with different contexts eliciting distinct types of MST. Parental and child MST were associated, though not linked to children’s task performance. Findings highlight the importance of diverse interaction contexts and suggest a need for longitudinal research on MST’s developmental impact.

## 1. Introduction

Early parent–child interactions play a crucial role in shaping children’s linguistic and social–emotional development (e.g., Devine & Hughes, 2018; Eisenberg et al., 1998; Tamis-LeMonda et al., 2019). These interactions include both verbal exchanges and non-verbal exchanges. Among verbal exchanges, mental state talk (MST) has been identified as particularly important for nurturing children’s developing social–emotional skills (Brownell et al., 2013; Eisenberg et al., 1998; Tompkins et al., 2018). MST is defined as the vocabulary that is used to refer to people’s state of mind: their thoughts, feelings, intentions, and so on. Consider the following example of a conversation between a father and his three-year-old daughter while grocery shopping.

Parent: “What’s your favorite food?”

Child: “Pancakes!”

Parent: “Mmm, pancakes are yummie! I like them too. But guess what—my favorite food is something else. Can you guess?”

Child: “Pizza?”

Parent: “That’s a good guess! I think you said pizza because it’s your other favorite. But no, my favorite is rice with lots of veggies.”

Child: “Eww, veggies are gross.”

Parent: “I know you feel that way. But veggies help you grow strong—like Daddy! And guess what? We’re having my favorite for dinner tonight—rice with lots of veggies.”

Child (frowning): “I want pizza.”

Parent: “I hear you, you’re feeling disappointed, huh? It’s okay to feel that way. We’ll have pizza another day. Tonight, it’s Daddy’s turn to choose.”

In this example, the father referred to his own and his daughter’s mental states on multiple occasions. He contrasted his daughter’s favorite food to his own—thereby communicating that their tastes and preferences differ. He also labelled her emotional reaction to the fact that she could not have pizza for dinner. Mental states are invisible, abstract constructs that must be inferred from contextual cues, such as facial expressions or behavior (i.e., taking a cookie implies wanting it). Through MST, parents scaffold their children’s ability to make such inferences independently—a skill commonly referred to as Theory of Mind (ToM) (Devine & Hughes, 2018; Tompkins et al., 2018; Wellman, 2018). However, engaging in MST can be particularly challenging for hearing parents of deaf or hard-of-hearing (DHH) children. The vast majority of these parents lack prior experience with DHH individuals and may feel insecure about how to communicate naturally with their child. As parental MST plays a pivotal role in fostering children’s social–emotional growth, gaining deeper insight into how hearing parents of young DHH children engage in MST is essential—especially given that DHH children are at heightened risk for experiencing challenges in their ToM and broader social–emotional development (Hoffman et al., 2016; Ketelaar et al., 2012; Netten et al., 2017; Pluta et al., 2021; Stevenson et al., 2015).

### 1.1. MST in Hearing Parents of DHH Children

To date, few studies have investigated the use of MST by hearing parents of DHH children. With the exception of a study by Pluta et al. (2023), existing research consistently indicates that hearing parents of DHH children refer to mental states less frequently in their interactions than hearing parents of hearing children (Dirks et al., 2020; Lind-Combs & Holt, 2022; Moeller & Schick, 2006; Morgan et al., 2014; Walker et al., 2017). The children in these studies ranged in age from 1 to 10 years, suggesting that this pattern is robust across a broad developmental span. Reported proportions of MST—or those calculated based on reported total word count and MST count—varied considerably across studies. For example, Dirks et al. and Morgan et al. reported relatively low proportions, ranging from 0.8% to 2.8%, whereas Walker et al. reported a higher proportion of 6%. The reasons for these discrepancies remain unclear. Age may be a contributing factor, but differences in MST coding systems across studies may also play a role.

One explanation for the finding that hearing parents of DHH children tend to use less MST than hearing parents of hearing children is that parents naturally tailor their MST to match their children’s developmental stage. Previous research has shown that parents of typically developing hearing children reference desires and emotions earlier than cognitive processes in their conversations with young children (Ensor et al., 2014; Ensor & Hughes, 2008; Taumoepeau & Ruffman, 2006). This pattern reflects the typical trajectory of ToM development, in which children first develop an understanding of emotions and desires before acquiring the ability to comprehend beliefs. Extending this, Lind-Combs and Holt (2022) and Morgan et al. (2014) both found that hearing parents of DHH children referred to desires and emotions as frequently as parents of hearing children of the same age but made fewer references to cognitive processes. Both studies suggest this may stem from parents’ perceptions of their DHH children’s language level, prompting less complex parental MST. In addition, Moeller and Schick (2006) reported that parents of DHH children used less varied MST—employing a smaller range of unique MS terms than parents of hearing children. In sum, existing research suggests that hearing parents of DHH children may produce a lower overall quantity of MST and use MST that is less complex and less varied, indicating potentially reduced quality compared to parents of hearing children.

At present, the study by Dirks et al. (2020) is the only one to examine MST use by both hearing parents and their young DHH children. Consistent with findings on parental MST, the study found that DHH children referred to mental states less frequently than their hearing peers. Moreover, a strong association was observed between children’s and parents’ use of MST, suggesting that parental input may play a significant role in shaping DHH children’s MS language, as it does in hearing children (Ruffman et al., 2002).

### 1.2. Storybook Reading Versus Play Activities

The studies involving hearing parents of DHH children conducted to date have employed a variety of activities to assess parental MST. These activities have included storybook reading (Pluta et al., 2023), free play (Dirks et al., 2020; Lind-Combs & Holt, 2022; Moeller & Schick, 2006), discussing art (Walker et al., 2017), and sharing family photos and watching and discussing movie scenarios (Moeller & Schick, 2006). The type of activity used to assess parental MST may affect both the quantity and the quality of MST observed. While differences between activities have not yet been examined in hearing parents of DHH children, some studies have explored this topic in hearing parents of hearing children. Studies that compared storybook reading (or storytelling) with free play showed that parents used more MST during storybook reading than during interactions involving play (Drummond et al., 2014; Farkas et al., 2018).

Children’s storybooks are typically rich in mental state vocabulary, offering children exposure to terms related to thoughts, feelings, and intentions (Cassidy et al., 1998; Dyer et al., 2000). Simply reading storybooks allows parents to introduce this vocabulary in a natural and engaging context. When parents go beyond simply reading—by asking open-ended questions and actively involving their children in discussions about characters’ facial expressions, the causes of their emotions, and the intentions, desires, and beliefs behind their actions—they further support the development of children’s social–emotional understanding (Adrian et al., 2005; Ebert et al., 2020; Schapira & Aram, 2020; Tompkins et al., 2022). Using a wordless picture book instead of a traditional storybook with text encourages parents to initiate conversations about characters’ inner states. Ziv et al. (2013) found that mothers’ MST was of higher quality with a wordless picture book compared to reading a traditional storybook.

Semi-structured play activities—in which parents are instructed to play with their child as they normally would, but with a preselected set of toys—have been used in multiple studies with hearing parents and hearing children as a means to observe MST (e.g., Colonnesi et al., 2019; Devine & Hughes, 2019; Laranjo et al., 2014). Given that young children spend a lot of their waking time engaged in play (Soderstrom & Wittebolle, 2013) and that parents from industrialized societies generally value and enjoy playing with their children (Lansford, 2021), free play with toys provides an ecologically valid setting to assess MST.

### 1.3. Parental MST and Children’s Social–Emotional Competence

Very few studies thus far have investigated the role of parental MST in relation to DHH children’s social–emotional competence. While Dirks et al. (2020) found that parental MST was associated with DHH children’s own use of MST, other studies (i.e., Moeller & Schick, 2006; Pluta et al., 2023; Walker et al., 2017) have shown an association with broader aspects of DHH children’s social–emotional competence. Pluta et al. examined parental MST during storybook reading in association with ToM abilities in 39 deaf children with cochlear implants (3 to 8 years old). Uniquely, they assessed ToM using a parent-report questionnaire rather than direct child assessments. Their findings indicated positive associations between parental MST—specifically references to emotions and cognitive processes—and aspects of children’s ToM abilities. Similarly, Moeller and Schick found that overall maternal MST was related to ToM performance in 22 deaf children aged 4–10 years, although they did not distinguish between different types of MST. Their sample included 10 children using cochlear implants, 10 using hearing aids, and two who did not use any form of hearing technology. In contrast to these cross-sectional designs, Walker et al. conducted the only longitudinal study on this topic. In a sample of 46 hard-of-hearing children, they found that parental MST measured at age three predicted ToM abilities at age five.

While studies involving DHH populations are still emerging, more extensive evidence is available for hearing parents and their hearing children. A meta-analysis by Tompkins et al. (2018), which synthesized findings from 40 studies involving 2298 children, found that parental MST was positively associated with children’s emotion understanding and ToM—specifically their understanding of false beliefs (FB). Stronger relations were observed for references to cognitive processes than for references to emotions or desires. Devine and Hughes (2018) also conducted a meta-analysis examining links between parental MST and children’s FB understanding in 28 studies, involving 1914 three- to five-year-old children. They too concluded that parental MST was positively associated with FB understanding.

### 1.4. The Present Study

Although several studies have examined how hearing parents of DHH children use MST, the evidence remains limited and fragmented. Most research has focused on the quantity of MST, showing that parents of DHH children produce fewer mental state references than parents of hearing children (Dirks et al., 2020; Lind-Combs & Holt, 2022; Moeller & Schick, 2006; Morgan et al., 2014; Walker et al., 2017). Only three of these studies also addressed the quality of MST, with findings suggesting that it may also differ (Lind-Combs & Holt, 2022; Moeller & Schick, 2006; Morgan et al., 2014). However, none of these studies have contrasted different types of activities, leaving open the question of how MST may vary across different contexts, such as storybook reading versus free play. Another notable gap is the limited attention to the MST of DHH children themselves. Furthermore, the relationship between parental MST and DHH children’s social–emotional functioning—including children’s own use of MS vocabulary—remains poorly understood. This is particularly relevant given that DHH children often show delays in social–emotional development compared to their hearing peers (e.g., Ketelaar et al., 2012; Li et al., 2024; Netten et al., 2017; Pluta et al., 2021; Stevenson et al., 2015).

Together, these gaps underscore the need for a more comprehensive investigation of MST in families with DHH children. The present study addresses this need by examining both the quantity and quality of parental MST across different interaction contexts, as well as exploring its relationship with children’s own MST and social–emotional functioning. Therefore, the following research questions and corresponding hypotheses were formulated for this study:

1. How much MST do hearing parents of DHH children use, and does this vary across activities (storybook reading versus free play)? We had no specific expectation regarding the quantity of MST in general, but we did expect that parents would use more MST during storybook reading than during free play;
2. Is parents’ overall use of MST more varied (i.e., of higher quality) during storybook reading than during free play? Given our expectation that parents would use more MST during storybook reading than during free play, we further hypothesized that they would also demonstrate a greater variety of MST in storybook reading;
3. Do parents of DHH children use more MST in each of four distinct categories (feeling states, cognitive processes, desires/value judgments, perceptions) during storybook reading or during free play? Previous research has shown activity-specific differences in MST use. Drummond et al. (2014) found that parents of hearing children referred more to emotions during storybook reading but more to cognitive processes and desires during free play. Similarly, in their longitudinal study, Farkas et al. (2018) reported more emotion references during storytelling and more desire references during free play at both time points. However, differences in references to cognitive process were only observed when children were 10–15 months old— favoring storybook reading—and not when they were 28–33 months old. Based on these findings, we expected parents to use more references to feeling states during storybook reading and more desire references during free play. No specific expectation was formulated for cognitive processes due to inconsistent findings across studies, nor for references to perceptions, as this category was not examined in either study;
4. Is parents’ use of MST in each of four distinct categories more varied during storybook reading or during free play? Farkas et al. (2018) was the only study to also examine variation within specific MST categories. Their findings mirrored those for quantity: parents showed greater diversity in emotion references during storytelling and greater diversity in desire references during free play. However, results for cognitive processes were inconsistent, with more variation observed during storytelling only when children were 10–15 months old and not when they were 28–33 months old. Based on these findings, we expected parents to use more varied references to feeling states during storybook reading and more varied references to desires during free play. No specific expectations were formulated for cognitive processes due to inconsistent findings, nor for references to perceptions, as this category was not examined in the cited study;
5. How much MST do DHH children use, and does their use of MST differ across activities? We had no specific expectations regarding the quantity of MST in general or regarding differences in MST between the two activities;
6. Is DHH children’s overall use of MST more varied during storybook reading or during free play? No expectation was formulated regarding variation in DHH children’s MST;
7. Do DHH children use more MST in each of four distinct categories during storybook reading or during free play? No expectations were formulated regarding this research question;
8. Is DHH children’s use of MST in each of four distinct categories more varied during storybook reading or during free play? No expectations were formulated regarding this research question;
9. How is the quantity of parental MST in each of four distinct categories related to DHH children’s social–emotional competence—including their MST—and demographic factors, such as age and degree of hearing loss? We expected that parental MST would be positively associated with social–emotional competence and MST in DHH children. Additionally, it was expected that parents with older children would refer more often to cognitive processes than parents with younger children;
10. How is child MST related to children’s social–emotional competence and demographic factors? We hypothesized that child MST would be positively associated with children’s performance on the social–emotional tasks and with their MS vocabulary as reported by parents. Furthermore, we expected older children to refer more often to cognitive processes.

## 2. Materials and Methods

### 2.1. Participants

Participants were recruited from six organizations for FCEI (family-centered early intervention) in the Netherlands. In total, 48 hearing parent–DHH child dyads participated in this study. Inclusion criteria were current enrollment in FCEI and, for the participating children, a permanent bilateral, unaided hearing loss of >35 dB in the better ear. Additionally, parents were required to be proficient in either spoken Dutch, Sign Language of the Netherlands (NGT), or Sign-Supported Dutch (NmG). In the vast majority of children (*n* = 45, 94%), hearing loss was detected through newborn hearing screening. In two children, hearing loss was detected between 1;0 and 1;6. For one child, the age at which hearing loss was detected was unclear, but detection occurred before the age of 3 years. Table 1 shows characteristics of the sample.

### 2.2. Procedure

This study was part of a larger, longitudinal intervention study investigating the effectiveness of a parent program designed to support parents in using more MST with their young DHH children. Approval for the study was granted by Utrecht University’s Ethics Committee (no. 21-0209).

The data presented here were collected prior to parents’ participation in the program. Data was collected during home visits. Researchers experienced in administering tasks to DHH children were trained by the first author to conduct these home visits as per the protocol written for this study. Researchers were proficient in the child’s preferred mode of communication—spoken Dutch, NGT, or NmG—and used it consistently throughout the home visit, including during task administration. All sessions were recorded on video.

After getting acquainted with the child and parent, the researcher invited the parent to engage in storybook reading with their child as they normally would, using a wordless picture book selected for the study (“The umbrella” by Ingrid and Dieter Schubert). This book features a small dog who finds an umbrella. The umbrella catches a gust of wind, taking the dog on an adventurous journey around the world. After five minutes, the parent was cued by the researcher to wrap up the story and switch to the next activity. The dyads engaged in free play for another five minutes with a fixed set of toys (i.e., tea set, wooden block puzzle, and animal figures). It was up to the dyads to choose which toy(s) to play with and switching between toys was allowed.

Subsequently, the researcher administered four tasks to measure children’s emotion and ToM understanding. The order of the four tasks was counterbalanced across participants, but the emotion-identification task always preceded the emotion-attribution task. If children were unable to accurately identify the emotions in the identification task, the attribution task was omitted, as it relied on foundational emotion-identification skills.

Additionally, parents filled out questionnaires about their family background (e.g., their educational level, home language(s), children’s age, gender, and audiological characteristics) and about their child’s mental state vocabulary. At the end of the visit, children received a small present as a token of appreciation for their participation.

### 2.3. Materials

#### 2.3.1. MST During Parent–Child Interaction

MST of parents and children was observed during two types of activities (storybook reading and free play). Language during these activities was transcribed and coded using Computerized Language Analysis (CLAN) software (V 01-Mar-2023 11:00), and analyzed with Codes for the Human Analysis of Transcripts (CHAT) (MacWhinney, 2000). All interactions were either in spoken language or sign-supported language. Utterances in both modalities were transcribed, and all MST was subsequently coded—for parents and children separately and for the two activities separately.

A coding scheme^1^ for coding MST was developed based on existing literature (Adrian et al., 2005; Doan & Wang, 2010; Jakubowska et al., 2018; Lind-Combs & Holt, 2022; Taumoepeau & Ruffman, 2006). MS terms were categorized into one of four categories: references to *feeling states* (e.g., happy, mad, thankful), *cognitive processes* (e.g., think, understand, believe), *desires/value judgments* (e.g., want, prefer, stupid), and *perceptions* (e.g., see, look, listen). If an MST word was supported by sign, this was coded as a single MST instance.

A group of eleven coders, with backgrounds in linguistics or psychology, coded MST from the video recordings. Prior to coding the data, they were trained by the first and second author to familiarize them with the coding scheme and to practice coding according to the coding scheme. Throughout the coding phase, all coders notified the first and second author if they came across a word that they identified as an MS word—fitting into one of the four previously identified categories—that was not included in the coding scheme. If deemed appropriate by the first and second author, the word was added to the scheme, and all coders were made aware of the new addition. At the end of the coding phase, all coded transcripts were reviewed to code these new additions.

To determine intercoder reliability, a master coder (the second author) double-coded 20% of the transcripts. These transcripts were equally distributed across coders, participants and activities (storybook reading and free play). The statistical package K-Alpha Calculator (Marzi et al., 2024) was used to calculate Krippendorff’s Alpha (Krippendorff, 2019) as an estimate of intercoder reliability. Intercoder reliability values ranged between 0.958 and 1.000 with an overall value of 0.997, which represents highly satisfactory levels of agreement coming in well above Krippendorff’s (2019) suggested threshold of 0.80. Additionally, we determined inter-coder agreement by calculating the percentage of agreement. Given that missing values were the most prone mistake in coding the transcripts and that missing data impact percentages more than they impact Krippendorff’s Alpha, reporting percentages provides a more conservative measure of agreement. Percentage of agreement for each individual coder with the master coder ranged from 83% to 100% with a mean of 94%.

MST was calculated in two ways. First, the total number of mental state terms uttered was counted, per category and overall, in order to obtain measures of quantity. Second, the number of unique mental state terms was counted, per category and overall, in order to obtain measures of quality. To clarify, within the category feeling states the three mental state words scared, happy, and happy represent three instances of MST (quantity count), but there are only two unique words (quality count). To calculate the proportion of MST, the total number of MS words, per category and overall, was divided by the total word count. Five recordings (two for storybook reading and three for free play) were shorter than the required five minutes. This was normalized by multiplying all counts by a factor based on the actual duration of each recording versus the required duration, so the counts reflected the language that would have been produced in five minutes.

#### 2.3.2. Mental State Vocabulary 

To obtain a complementary perspective in addition to the direct observation of MST during the two activities, parents reported on their children’s mental state vocabulary by indicating whether their child generally understood and used each of 20 mental state words (e.g., happy, sad, think, dream, want) in everyday life (0 = no, 1 = yes) (Ketelaar et al., 2015). This parent-report measure captures a broader and more habitual picture of children’s MST across daily contexts. Mean scores were calculated across all items, resulting in a score (proportion) between 0 and 1. For example, when a child understood and used 5 out of 20 words, this resulted in a score of 0.25.

#### 2.3.3. Emotion Identification 

The Emotion-Identification Task (Wiefferink et al., 2013) was conducted to examine whether children were able to recognize the four basic emotions: happiness, anger, sadness, and fear. The children were shown eight drawings of facial emotion expressions, two per emotion. They were asked to point at the corresponding facial expression when asked: “Who looks [happy/angry/sad/scared]?”. If children only pointed to one face, the researcher asked: “Does anyone else look [happy/angry/sad/scared]?”. Children were awarded one point for each correctly identified facial expression. A mean score ranging between 0 and 2 was calculated across the four emotions.

#### 2.3.4. Emotion Attribution 

The Emotion-Attribution Task (Wiefferink et al., 2013) aims to assess children’s ability to attribute emotions based on situational context. The children were shown eight illustrations depicting prototypical emotion-eliciting scenarios. The researcher neutrally described each situation (e.g., “Look, the boy’s tower is knocked over”) while showing the corresponding illustration and asked the child how the protagonist would feel (verbal condition). After the child responded, the researcher revealed four facial expressions (happiness, sadness, anger, and fear) and asked what the protagonist’s face would look like (visual condition). Responses were scored separately for each condition. A response was considered correct if the child attributed an emotion within the intended valence domain—positive (happiness) or negative (anger, sadness, fear)—to account for variability in emotional interpretation. For example, the protagonist might feel sad about the loss, angry at the perpetrator, or fearful of aggressive behavior. Children received one point per correct response, and scores were calculated as proportions of correct answers per condition.

#### 2.3.5. Desires 

To assess children’s understanding of others’ divergent preferences, we used the Uncommon-Desire Task (Ketelaar et al., 2012). Children were presented with two short stories, each accompanied by two illustrations. The first illustration showed two food items—ice cream and tomato in the first story, cake and carrot in the second—and children were asked which item they preferred. In the second illustration, a boy was added, and children were told that he dislikes the child’s preferred item but likes the other one. Children were then asked a test question (“Which of these food items will the boy pick?”) and two control questions (“Does the boy like [child’s preferred item]?” and “Does the boy like [child’s non-preferred item]?”. To pass the task, children had to answer all three questions correctly (0 = fail, 1 = pass). Mean scores were calculated across the two stories.

#### 2.3.6. False Belief 

To assess children’s understanding of others’ false beliefs, we used a change-of-location false-belief task (Ketelaar et al., 2012). Children were told a short story supported by illustrations. In the story, a boy places his toy airplane in one location and then leaves the scene. While he is away, a girl moves the toy to a second location. Upon the boy’s return, children were asked a test question (“Where will the boy look for his plane?”) and two control questions (“Where is the plane now?” and “Where did the boy put the plane before he left?”). Children passed the task only if they answered all three questions correctly (0 = fail, 1 = pass).

### 2.4. Statistical Analyses

Data were analyzed using IBM SPSS Statistics, version 27. First, paired samples *t*-tests were conducted to compare the two activities—storybook reading and free play—in terms of both the quantity and quality of MST. The Holm correction method was applied to adjust for multiple comparisons and reduce the risk of false positives. Next, correlational analyses were conducted, again using the Holm method, to examine relationships between MST and children’s social–emotional development and demographic characteristics. Partial Pearson correlations, controlling for age, were used to assess the association between parental MST and children’s social–emotional competence, given that both are expected to increase with age. Pearson and Spearman correlation analyses were conducted to examine associations between parental MST and demographic variables, including children’s age and degree of hearing loss. Similarly, partial Pearson correlations were used to assess the relationship between child MST and children’s social–emotional competence, while Pearson and Spearman correlations were applied to examine associations between child MST and demographic factors.

## 3. Results

### 3.1. Research Questions 1 and 2: Quantity and Quality of Parental MST

MST accounted for approximately 8% of parents’ total language use in both activities (storybook reading: 8.2%, free play: 7.9%). Parents spoke and/or signed more during storybook reading (M = 358.22, SD = 98.24) than during free play (M = 269.05, SD = 94.01), *t*(47) = 7.59, *p* < 0.001, *d* = 1.10. They also produced more MS words during storybook reading (*M* = 28.85, *SD* = 13.92) than during free play (*M* = 21.09, *SD* = 10.30), *t*(47) = 3.79, *p* < 0.001, *d* = 0.55). However, parents did not use more varied MST during storybook reading (*M* = 9.45, *SD* = 4.31) than during free play (*M* = 8.91, *SD* = 3.67), *t*(47) = 1.30, *p* = 0.200, *d* = 0.19.

To summarize, the quantity of parental MST was higher during storybook reading than during free play, but the quality—expressed as variation in MS words—did not differ significantly between activities.

### 3.2. Research Question 3 and 4: Quantity and Quality of Parental MST Across Four Distinct Categories

A series of paired *t*-tests to examine differences in quantity between storybook reading and free play for each of the four categories of MST revealed that parents referred more to feeling states, cognitive processes, and perceptions during storybook reading than during free play. Conversely, they referred more to desires/value judgments during free play than during storybook reading (see Table 2).

Similarly, paired *t*-tests were conducted to examine differences in quality between activities for the four MST categories. Significant differences were found for all categories except for cognitive processes (see Table 3). Parents used more varied references to feeling states and perceptions during storybook reading, and to desires/value judgments during free play.

### 3.3. Research Questions 5 and 6: Quantity and Quality of Child MST

Across both activities, MST accounted for 5.4% of children’s language. Although MST made up a slightly larger proportion of children’s utterances during storybook reading (6.1%) than during free play (4.6%), this difference was not statistically significant, *t*(47) = 1.70, *p* = 0.096, *d* = 0.25. Children did not differ significantly in total word count between storybook reading (*M* = 75.03, *SD* = 52.83) and free play (*M* = 70.39, *SD* = 52.83), *t*(47) = 0.96, *p* = 0.340, *d* = 0.14. However, they produced more MS words during storybook reading (*M* = 4.81, *SD* = 5.11) than during free play (*M* = 3.42, *SD* = 3.97), *t*(47) = 2.07, *p* = 0.044, *d* = 0.30. Children did not use more varied MST during storybook reading (*M* = 1.92, *SD* = 1.90) than during free play (*M* = 1.84, *SD* = 1.71), *t*(47) = 0.36, *p* = 0.721, *d* = 0.05.

To summarize, the quantity of child MST was higher during storybook reading than during free play, whereas the quality—expressed as variation in MS words—did not significantly differ between activities.

### 3.4. Research Questions 7 and 8: Quantity and Quality of Child MST Across Four Distinct Categories

Paired samples *t*-tests were conducted to examine differences in the quantity of MST across the two activities—storybook reading and free play—for each of the four MST categories. Results indicated that children made more references to feeling states and perceptions during storybook reading than during free play. Conversely, references to desires/value judgments were more frequent during free play than during storybook reading (see Table 4).

A second set of paired *t*-tests was conducted to assess differences in the quality of children’s MST across activities, again for each MST category. These analyses revealed that children produced a more varied range of references to feeling states during storybook reading, whereas references to desires/value judgments were more varied during free play (see Table 5).

### 3.5. Research Question 9: Correlations Between Parental MST and Children’s Social–Emotional Competence and Demographic Factors

There was a significant one-tailed partial correlation, controlling for age, between the total quantity of parental MST and the total quantity of child MST collapsed across the two activities, *r* = 0.40, *p* = 0.003. Parental MST was also positively correlated with children’s parent-reported MS vocabulary, *r* = 0.27, *p* = 0.033. Table 6 shows one-tailed partial correlations, controlling for age, between the four categories of parental MST and children’s performance on the social–emotional tasks. After Holm correction was applied, none of the correlations remained significant.

Of the four categories of parental MST, only parents’ references to cognitive processes showed a significant one-tailed correlation with children’s age, *r* = 0.36, *p* = 0.006. The categories of feeling states (*r* = 0.21, *p* = 0.07), desires/value judgments (*r* = 0.20, *p* = 0.085), and perceptions (*r* = 0.02, *p* = 0.434) were not significantly correlated with children’s age. No significant correlation was found between total quantity of parental MST and children’s degree of hearing loss, *r* = 0.22, *p* = 0.105. Additionally, there was no significant difference in the quantity of parental MST between children with CI and children with HA (*t*(33) = −0.87, *p* = 0.392, *d* = −0.30).

### 3.6. Research Question 10: Correlations Between Child MST and Social–Emotional Competence and Demographic Factors

A significant one-tailed partial correlation, controlling for age, was found between child MST as observed during the parent–child activities and children’s MS vocabulary as reported by their parents, *r* = 0.32, *p* = 0.015. One-tailed partial correlations, controlling for age, between the four categories of child MST and children’s performance on the social–emotional tasks are reported in Table 7. None of these were significant. However, there was a significant one-tailed correlation between MS vocabulary and emotion identification.

Of the four categories of child MST, only children’s references to desires/value judgments showed a positive one-tailed correlation with age, *r* = 0.44, *p* < 0.001. One-tailed correlations between age and feeling states (*r* = 0.13, *p* = 0.188), cognitive processes (*r* = 0.23, *p* = 0.062), and perceptions (*r* = 0.20, *p* = 0.089) were all non-significant. No significant correlation was found between total quantity of child MST and children’s degree of hearing loss, *r* = 0.09, *p* = 0.300. Additionally, there was no significant difference in the quantity of child MST between children with CI and children with HA (*t*(33) = −0.20, *p* = 0.846, *d* = −0.07).

## 4. Discussion

This study aimed to contribute to a better understanding of parental MST and its relation to children’s social–emotional competence in hearing parent–DHH child dyads. The outcomes support the idea that hearing parents adapt their level of MST to their DHH children’s developmental abilities. Specifically, we found a positive association between children’s age and the frequency of parental references to cognitive processes, but not of feeling states, desires/value judgments, or perceptions. Talk about cognitions is generally considered more complex than talk about emotions or desires, which may explain why parents of older children—presumably with more advanced developmental skills—used more references to cognitive processes than parents of younger children. This aligns with previous research showing that parents tend to reference emotions and desires earlier than cognitive processes in conversations with their young hearing children (Ensor et al., 2014; Ensor & Hughes, 2008; Taumoepeau & Ruffman, 2006), and that hearing parents of DHH children refer less often to cognitive processes—but not to desires and emotions—than parents of hearing children (Lind-Combs & Holt, 2022; Morgan et al., 2014). Together, these findings suggest that parents tailor the complexity of their MST to match their child’s developmental level. Interestingly, children’s own references to cognitive processes were not related to age, whereas their references to desires/value judgments did show a positive association with age. This pattern may suggest that parents are offering MST that falls within the child’s *zone of proximal development* (Vygotsky, 1978)—providing input that slightly exceeds the child’s current level of competence and may support further growth.

No other associations with children’s characteristics emerged: the quantity of MST, whether produced by parents or children, was unaffected by children’s degree of hearing loss or by the type of device—HA or CI—they were using. We did not formulate specific expectations regarding these characteristics, in part because children with CIs—who typically have a more severe degree of hearing loss—may nonetheless have comparable or even better access to spoken language than children using hearing aids. However, these variables are often of interest in clinical and developmental research, and it remains important to examine whether and how they relate to outcomes such as MST. The lack of association in our findings suggests that parents may be responding more to observable developmental cues, such as age-related changes in communication and social–emotional milestones, than to audiological factors that may not directly reflect a child’s language access or social–emotional skills.

MST was observed during two ecologically valid activities: storybook reading and free play. As expected, and consistent with findings from studies involving hearing parent–hearing child dyads (Drummond et al., 2014; Farkas et al., 2018), parents of DHH children referred to MS more frequently during storybook reading than during free play. Across both activities, however, MST accounted for approximately 8% of parents’ total word use. Two aspects of these findings warrant attention.

First, the proportion of MST observed in this study is notably higher than those reported in previous research (Dirks et al., 2020; Morgan et al., 2014; Walker et al., 2017). This discrepancy may be attributable to differences in children’s age range, interaction context, or MST coding systems. In particular, earlier studies did not include the category perceptions—which encompasses terms such as look and see—and which accounted for nearly half of the MS references in the present study. Jakubowska et al. (2018) argue that beliefs often stem from perceptual experiences, and, therefore, perception terms constitute valid mental state language. This rationale informed our decision to include this category.

Second, parents were generally more talkative during storybook reading than during free play, and their MST increased proportionally. Although the relative proportion of parental MST did not differ between activities, children were exposed to a greater absolute number of MS references during five minutes of storybook reading than during five minutes of free play. Importantly, however, quantity alone does not capture the full picture—quality is equally, and arguably even more, important.

Variation, used here as an indicator of quality, was assessed in two ways. First, MST was categorized into four distinct types: feeling states, cognitive processes, desires/value judgments, and perceptions. In line with Drummond et al. (2014), our findings suggest that the content of parents’ MST was shaped by the activity in which they were engaged (see Table 2). Storybook reading prompted more questions and remarks about (the story character’s) cognitive processes and feelings—for example, “Do you *think* the dog is *scared*?”—as well as frequent references to perceptions, such as “*Look*, do you *see* the dog?”, typically used to direct the child’s attention to elements in the storybook.

In contrast, free play elicited more utterances related to desires/value judgments. Examples include “Which toy do you *want* to play with?” and “I’d *like* some tea please”. These references were often context-driven: parent–child dyads were handed three toy sets in one bag and had to decide which set to play with, prompting questions about preferences. One of the toy sets was a tea set, which encouraged imaginative play and talk about preferences regarding drinks and foods. As such, free play may be particularly conducive to eliciting references to desires and value judgments.

Second, we examined variation in the specific terms used within each MS category. Parents showed greater lexical diversity in their use of feeling state and perception terms during storybook reading, and in their use of desire/value judgment terms during free play (see Table 3). In other words, they repeated individual MS words less often, suggesting a richer and more varied MST vocabulary depending on the activity. These findings highlight the complementary nature of different parent–child activities, each eliciting distinct types of MST. Although storybook reading prompted a higher overall number of MS references, we do not advocate prioritizing this activity exclusively to support children’s development. Rather, we recommend that parents engage in a variety of activities that they and their child enjoy and feel comfortable with. A diverse range of interactions is likely to expose children to a broader spectrum of MST, which may be beneficial for their development. That said, when parents do engage in book-reading activities, we encourage the use of wordless storybooks, as these are particularly effective in stimulating MST-rich conversations between parent and child (Ziv et al., 2013).

In this study, we also examined DHH children’s use of MST—an area that, to date, has only been explored by Dirks et al. (2020). The pattern observed in DHH children closely mirrored that of their parents. Like their parents, children used MST more frequently during storybook reading than during free play, even though—unlike their parents—they did not produce more language overall in the storybook reading context. One possible explanation is that children’s increased use of MST during this activity was influenced by the higher number of MS references made by their parents. In terms of quality, storybook reading elicited more frequent and more varied references to feeling states, as well as more frequent references to perceptions. In contrast, free play prompted more frequent and more varied references to desires/value judgments (see Table 4 and Table 5).

Previous studies with hearing children have emphasized the importance of parental MST for children’s social–emotional development (Devine & Hughes, 2018; Eisenberg et al., 1998; Tompkins et al., 2018). For DHH children, exposure to high-quality and high-quantity MST may be especially valuable, given the distinct challenges they often face in developing social–emotional competencies (Hoffman et al., 2016; Ketelaar et al., 2012; Li et al., 2024; Pluta et al., 2021; Stevenson et al., 2015; Wiefferink et al., 2012, 2013). The present study is among the first to examine this relationship in DHH children. As expected, and consistent with Dirks et al. (2020), we found an association between parents’ and children’s use of MST. This association was evident both in the observed parent–child interactions and in parents’ general reports of their children’s MST use across everyday contexts. Again, this supports the notion that parents are attuned to their children’s MS language abilities (Taumoepeau & Ruffman, 2006). It should be noted, however, that because the data in this study are cross-sectional, the direction of the relationship remains unclear. It is possible that children’s MST reflects the input they receive from their parents, but it is equally plausible that parents adjust their MST in response to their children’s developing abilities—or that both processes occur simultaneously.

Contrary to expectations, however, no significant relationships emerged between parental MST observed during parent–child interaction and children’s performance on social–emotional tasks (see Table 6). One possible explanation for this is that the current study examined only concurrent associations. For example, Walker et al. (2017) found in their longitudinal study that parental MST predicted later social–emotional outcomes in hard-of-hearing children. Similarly, Tompkins et al. (2018) in their meta-analysis, argued that the effects of parental MST on children’s social–emotional development may emerge over time as children internalize and apply what they learn from MS-rich conversations. The present study is part of a larger, ongoing longitudinal intervention project with the same sample of parent–child dyads, which will allow us to further investigate these potential delayed effects; a manuscript on this broader project is in preparation.

Nevertheless, some previous cross-sectional studies have reported associations between parental MST and ToM abilities in deaf children (Moeller & Schick, 2006; Pluta et al., 2023). It is important to note, however, that the children in those studies were substantially older than those in the present sample. Specifically, the age ranges were three to eight years (Moeller & Schick, 2006) and four to ten years (Pluta et al., 2023), whereas our study included children between two and five years old. Thus, another possible explanation for the lack of associations in our study is the younger age of our participants. Indeed, children’s average scores on some social–emotional tasks—particularly the false belief task—were quite low, making it statistically challenging to detect relationships with parental MST.

Furthermore, we examined concurrent associations between children’s MST and their social–emotional competence (see Table 7). Similar to the findings for parental MST, no significant associations emerged between children’s MST—observed during parent–child interactions—and their performance on social–emotional tasks. However, children’s MS vocabulary, as reported by parents, was positively associated with children’s ability to identify emotions in drawings. This finding reinforces the earlier explanation that the lack of observed associations may be due to the cross-sectional nature of the data. It is possible that MST observed in a short-term interaction context does not fully capture children’s abilities, whereas parent-reported MS vocabulary reflects children’s language use across a longer time span and a wider range of contexts.

### Limitations and Future Directions

Although a relatively large number of families with very young DHH children participated in this study, the sample may not fully represent the broader DHH population, which is known to be highly heterogeneous (van der Zee & Dirks, 2022). We aimed to recruit a diverse participant group, but some limitations remain.

First, no children with deaf parents or parents who use sign language as their native language participated in the study. As a result, our findings are limited to hearing parents who communicated with their children using spoken language, sign-supported speech, or a combination of both. Previous research suggests that social–emotional development, and particularly ToM, may be unaffected in deaf children from deaf families (e.g., Courtin, 2000; Schick et al., 2007), possibly as a result of early and fluent access to MST in sign language. Studying MST in deaf families would therefore be highly valuable, as it could help explain why previous studies (e.g., Dirks et al., 2020; Morgan et al., 2014) have found lower levels of MST in hearing parents of DHH children. Second, the participating parents had a relatively high level of education. Third, although both mothers and fathers were included, 94% of participating parents were mothers. These patterns are common in developmental research, but they still limit generalizability. Prior studies (e.g., Ebert et al., 2017; Roby & Scott, 2022) suggest that parental MST and its relationship with children’s ToM may be influenced by socioeconomic status, of which education level is one component. Moreover, some evidence (e.g., Jenkins et al., 2003) suggests that MST may differ between mothers and fathers, although research in this area remains scarce. Future studies should aim to include more diverse families—including deaf parents, sign language users, fathers, and families from a broader range of socioeconomic contexts. Such diversity would allow for a more comprehensive understanding of how MST functions across different environments and how it supports social–emotional development in DHH children.

In addition, although our data were largely complete, not all families provided background information on child and family characteristics. Unlike the MS vocabulary questionnaire, which was completed during the home visit, the background questionnaire was sent online and had a lower response rate. Future studies should aim to collect more comprehensive background data—ideally during in-person sessions—to enable a fuller understanding of how family and child characteristics relate to MST use and social–emotional development.

Additionally, MST word counts showed considerable variability across participants, as reflected in the wide ranges reported in Table 2, Table 3, Table 4 and Table 5. Such variability is to be expected, given the heterogeneity of the DHH population. Pending further research across a broader range of samples, findings should be interpreted with caution.

Finally, MST was observed during two short (semi-)structured activities. It is important to acknowledge that five minutes of storybook reading and another five minutes of free play—with an experimenter present and video recording the session—may not accurately reflect parents’ and children’s typical use of MST in everyday life. Future studies should consider exploring MST across multiple naturalistic contexts and over longer periods of time. Employing less conspicuous recording equipment capable of capturing an entire day’s routines and activities, along with automated methods to facilitate the analysis of parent–child interactions within them, could also help provide an even more ecologically valid picture of MST use in daily life.

## 5. Conclusions

In conclusion, this study contributes to the growing body of research on MST by examining both the quantity and quality of MST in a relatively large sample of hearing parents and their young DHH children, including children with varying degrees of hearing loss who used different types of hearing devices—primarily hearing aids or cochlear implants. It is among the first to explore the relationship between MST and social–emotional competence in DHH children, and the first to compare MST across two naturalistic parent–child activities—storybook reading and free play—in this population.

Our findings show that hearing parents adjust the complexity of their MST, suggesting sensitivity to developmental cues such as children’s age, albeit not their audiological characteristics. Moreover, the results underscore the importance of engaging in a variety of interaction contexts, as different activities elicit distinct types of MST. Parents should be supported in recognizing and using everyday moments—such as playtime and shared book reading, mealtime, and bedtime—as opportunities to talk about thoughts, feelings, and desires.

Although we found associations between parental MST and children’s MST, these were not reflected in children’s performance on social–emotional tasks. This may be due to the cross-sectional nature of the data and the young age of the children, whose social–emotional skills are still emerging. It is likely that the effects of MST on children’s development unfold over time, as children internalize and apply MS language in diverse contexts. Therefore, longitudinal research is needed—particularly in young DHH children—to better understand how MST exposure contributes to social–emotional development.

## Figures and Tables

**Table 1:** Characteristics of the sample.

**Number of parent–child dyads (N)**	48
**Participating parent sex, n (%)**	
Mother	44 (92%)
Father	4 (8%)
**Parental education level** ^a^**, n (%)**	
Secondary vocational	10 (20%)
Higher professional/College	16 (33%)
University	11 (23%)
Missing data	11 (23%)
**Child age, months**	
Mean (SD)	40.8 (8.5)
Range	27–64
**Child sex, n (%)**	
Male	23 (48%)
Female	25 (52%)
**Type of hearing device, n (%)**	
HA	24 (50%)
CI (+HA)	20 (42%)
BCD	2 (4%)
Missing data	2 (4%)
**Age at provision of first hearing device, months**	
Median (SD)	6 (8.8)
Range	3–35
Missing data, n	16
**Hearing age** ^b^**, months**	
Median (SD)	28.5 (9.4)
Range	10–54
Missing data, n	16
**Degree of hearing loss (best ear), n (%)**	
Moderate (36–50 dB)	9 (19%)
Moderately severe (51–65 dB)	9 (19%)
Severe (66–80 dB)	5 (10%)
Severe-to-profound (81–95 dB)	5 (10%)
Profound (>95 dB)	8 (17%)
Missing data	12 (25%)
**Child’s preferred communication mode(s), n (%)**	
Spoken	14 (29%)
Sign-supported	10 (21%)
Spoken & sign-supported	24 (50%)
**Home communication mode(s), n (%)**	
Spoken	12 (25%)
Sign supported	5 (10%)
Spoken & sign-supported	12 (25%)
Missing data	19 (40%)
**Social–emotional competence, mean (SD), range (min.–max.)**	
Mental state vocabulary (parent questionnaire)	0.46 (0.22), 0–1
Emotion identification (task)	1.10 (0.71), 0–2
Emotion attribution—verbal condition (task)	0.21 (0.32), 0–1
Emotion attribution—visual condition (task)	0.42 (0.41), 0–1
Desires (task)	0.39 (0.45), 0–1
False belief (task)	0.02 (0.14), 0–1

^a^ Parental education level represents the highest level of education achieved by either parent. ^b^ Hearing age is time since first device provision. Abbreviations: BCD = bone conduction device; CI = cochlear implant; HA = hearing aid; dB = decibel; SD = standard deviation.

**Table 2:** Quantity of parental MST (mean, SD, and range) during storybook reading and free play.

	Storybook Reading	Free Play	Test Statistic ^a^
Feeling states	1.69 (2.43), 0–13	0.82 (1.32), 0–6	*t*(47) = 2.39, *p_cor_* = 0.042, *d* = 0.35
Cognitive processes	5.80 (5.89), 0–33	4.59 (5.38), 0–23	*t*(47) = 2.17, *p_cor_* = 0.042, *d* = 0.31
Desires/value judgments	4.74 (3.14), 0–14	8.98 (5.05), 0–24	*t*(47) = −5.68, *p_cor_* < 0.001, *d* = −0.82
Perceptions	16.61 (8.11), 4–49	6.71 (6.17), 0–34	*t*(47) = 6.86, *p_cor_* < 0.001, *d* = 0.99

^a^ Paired samples *t*-tests, with Holm correction applied.

**Table 3:** Quality of parental MST (mean, SD, and range) during storybook reading and free play.

	Storybook Reading	Free Play	Test Statistic ^a^
Feeling states	1.23 (1.69), 0–9	0.61 (0.92), 0–3	*t*(47) = 2.37, *p_cor_* = 0.044, *d* = 0.34
Cognitive processes	3.06 (2.10), 0–10	2.51 (2.34), 0–10	*t*(47) = 1.96, *p_cor_* = 0.056, *d* = 0.28
Desires/value judgments	2.94 (1.44), 0–8	4.00 (1.80), 0–8	*t*(47) = −4.01, *p_cor_* < 0.001, *d* = −0.58
Perceptions	2.22 (0.64), 1–4	1.78 (0.73), 0–4	*t*(47) = 3.91, *p_cor_* < 0.001, *d* = 0.56

^a^ Paired samples *t*-tests, with Holm correction applied.

**Table 4:** Quantity of child MST (mean, SD, and range) during storybook reading and free play.

	Storybook Reading	Free Play	Test Statistic ^a^
Feeling states	0.60 (1.38), 0–7	0.08 (0.45), 0–3	*t*(47) = 2.43, *p_cor_* = 0.048, *d* = 0.35
Cognitive processes	1.00 (1.86), 0–7	0.87 (2.16), 0–10	*t*(47) = 0.54, *p_cor_* = 0.591, *d* = 0.08
Desires/value judgments	0.42 (0.92), 0–4	1.15 (1.52), 0–6	*t*(47) = −3.05, *p_cor_* = 0.016, *d* = −0.44
Perceptions	2.79 (4.02), 0–17	1.31 (1.74), 0–7	*t*(47) = 2.50, *p_cor_* = 0.048, *d* = 0.36

^a^ Paired samples *t*-tests, with Holm correction applied.

**Table 5:** Quality of child MST (mean, SD, and range) during storybook reading and free play.

	Storybook Reading	Free Play	Test Statistic ^a^
Feeling states	0.40 (0.71), 0–3	0.04 (0.20), 0–1	*t*(47) = 3.24, *p_cor_* = 0.008, *d* = 0.47
Cognitive processes	0.56 (0.99), 0–5	0.43 (0.84), 0–4	*t*(47) = 1.12, *p_cor_* = 0.534, *d* = 0.16
Desires/value judgments	0.31 (0.66), 0–3	0.68 (0.83), 0–3	*t*(47) = −3.13, *p_cor_* = 0.009, *d* = −0.45
Perceptions	0.65 (0.60), 0–2	0.69 (0.80), 0–3	*t*(47) = −0.31, *p_cor_* = 0.755, *d* = −0.05

^a^ Paired samples *t*-tests, with Holm correction applied.

**Table 6:** One-tailed partial correlations ^a^, controlling for age, between categories of parental MST and children’s social–emotional competence.

	Child	Emotion Identification	Emotion Attribution—Verbal Condition	Emotion Attribution—Visual Condition	Desires	False Belief
Parent						
Feeling states		−0.18	0.03	0.01	0.06	−0.14
Cognitive processes		0.14	0.36	0.18	0.29	−0.08
Desires/value judgments		−0.04	0.30	−0.06	0.13	0.10
Perceptions		−0.20	0.07	−0.11	−0.08	−0.02

* *p* < 0.05; ** *p* < 0.01; *** *p* < 0.001. ^a^ With Holm correction applied.

**Table 7:** One-tailed partial correlations ^a^, controlling for age, between categories of child MST and children’s social–emotional competence.

	Child	Emotion Identification	Emotion Attribution—Verbal Condition	Emotion Attribution—Visual Condition	Desires	False Belief
Child						
Feeling states		−0.20	−0.10	−0.07	−0.09	−0.11
Cognitive processes		0.19	−0.10	0.15	−0.10	0.02
Desires/value judgments		−0.10	0.21	−0.03	−0.18	−0.22
Perceptions		−0.10	−0.03	−0.09	0.00	−0.17
MS vocabulary		0.42 *	0.24	0.28	0.17	0.11

* *p* < 0.05; ** *p* < 0.01; *** *p* < 0.001. ^a^ With Holm correction applied.

## Data Availability

The dataset presented in this article is not readily available because the data are part of an ongoing study. Requests to access the dataset should be directed to the corresponding author.

## Note

1 Coding scheme available upon request from the corresponding author.

## Abbreviations

The following abbreviations are used in this manuscript:

MST	Mental state talk
ToM	Theory of mind
DHH	Deaf or hard of hearing
FB	False belief
FCEI	Family-centered early intervention
dB	Decibel
NGT	Dutch sign language
NmG	Sign-supported Dutch
HA	Hearing aid
CI	Cochlear implant
BCD	Bone conduction device

## References

[B1-behavsci-15-01558] Adrian J. E., Clemente R. A., Villanueva L., Rieffe C. (2005). Parent–child picture-book reading, mothers’ mental state language and children’s theory of mind. Journal of Child Language.

[B2-behavsci-15-01558] Brownell C. A., Svetlova M., Anderson R., Nichols S. R., Drummond J. (2013). Socialization of early prosocial behavior: Parents’ talk about emotions is associated with sharing and helping in toddlers. Infancy.

[B3-behavsci-15-01558] Cassidy K. W., Ball L. V., Rourke M. T., Werner R. S., Feeny N., Chu J. Y., Lutz D. J., Perkins A. (1998). Theory of mind concepts in children’s literature. Applied Psycholinguistics.

[B4-behavsci-15-01558] Colonnesi C., Zeegers M. A. J., Majdandžić M., van Steensel F. J. A., Bögels S. M. (2019). Fathers’ and mothers’ early mind-mindedness predicts social competence and behavior problems in childhood. Journal of Abnormal Child Psychology.

[B5-behavsci-15-01558] Courtin C. (2000). The impact of sign language on the cognitive development of deaf children: The case of theories of mind. Journal of Deaf Studies and Deaf Education.

[B6-behavsci-15-01558] Devine R. T., Hughes C. (2018). Family correlates of false belief understanding in early childhood: A meta-analysis. Child Development.

[B7-behavsci-15-01558] Devine R. T., Hughes C. (2019). Let’s talk: Parents’ mental talk (not mind-mindedness or mindreading capacity) predicts children’s false belief understanding. Child Development.

[B8-behavsci-15-01558] Dirks E., Stevens A., Kok S., Frijns J., Rieffe C. (2020). Talk with me! Parental linguistic input to toddlers with moderate hearing loss. Journal of Child Language.

[B9-behavsci-15-01558] Doan S. N., Wang Q. (2010). Maternal discussions of mental states and behaviors: Relations to emotion situation knowledge in European American and immigrant Chinese children. Child Development.

[B10-behavsci-15-01558] Drummond J., Paul E. F., Waugh W. E., Hammond S. I., Brownell C. A. (2014). Here, there and everywhere: Emotion and mental state talk in different social contexts predicts empathic helping in toddlers. Frontiers in Psychology.

[B11-behavsci-15-01558] Dyer J. R., Shatz M., Wellman H. M. (2000). Young children’s storybooks as a source of mental state information. Cognitive Development.

[B12-behavsci-15-01558] Ebert S., Lehrl S., Weinert S. (2020). Differential effects of the home language and literacy environment on child language and theory of mind and their relation to socioeconomic background. Frontiers in Psychology.

[B13-behavsci-15-01558] Ebert S., Peterson C., Slaughter V., Weinert S. (2017). Links among parents’ mental state language, family socioeconomic status, and preschoolers’ theory of mind development. Cognitive Development.

[B14-behavsci-15-01558] Eisenberg N., Cumberland A., Spinrad T. L. (1998). Parental socialization of emotion. Psychological Inquiry.

[B15-behavsci-15-01558] Ensor R., Devine R. T., Marks A., Hughes C. (2014). Mothers’ cognitive references to 2-year-olds predict theory of mind at ages 6 and 10. Child Development.

[B16-behavsci-15-01558] Ensor R., Hughes C. (2008). Content or connectedness? Mother-child talk and early social understanding. Child Development.

[B17-behavsci-15-01558] Farkas C., del Real M. T., Strasser K., Álvarez C., Santelices M. P., Sieverson C. (2018). Maternal mental state language during storytelling versus free-play contexts and its relation to child language and socioemotional outcomes at 12 and 30 months of age. Cognitive Development.

[B18-behavsci-15-01558] Hoffman M. F., Cejas I., Quittner A. L. (2016). Comparisons of longitudinal trajectories of social competence: Parent ratings of children with cochlear implants versus hearing peers. Otology and Neurotology.

[B19-behavsci-15-01558] Jakubowska J., Szpak M., Białecka-Pikul M. (2018). How do parents use mental state language during narration to their 2- and 4-year-old children?. Psychology of Language and Communication.

[B20-behavsci-15-01558] Jenkins J. M., Turrell S. L., Kogushi Y., Louis S., Ross H. S. (2003). A longitudinal investigation of the dynamics of mental state talk in families. Child Development.

[B21-behavsci-15-01558] Ketelaar L., Rieffe C., Wiefferink C. H., Frijns J. H. M. (2012). Does hearing lead to understanding? Theory of Mind in toddlers and preschoolers with cochlear implants. Journal of Pediatric Psychology.

[B22-behavsci-15-01558] Ketelaar L., Wiefferink C. H., Frijns J. H. M., Broekhof E., Rieffe C. (2015). Preliminary findings on associations between moral emotions and social behavior in young children with normal hearing and with cochlear implants. European Child and Adolescent Psychiatry.

[B23-behavsci-15-01558] Krippendorff K. (2019). Content analysis: An introduction to its methodology.

[B24-behavsci-15-01558] Lansford J. E. (2021). Cross-cultural similarities and differences in parenting. Journal of Child Psychology and Psychiatry, and Allied Disciplines.

[B25-behavsci-15-01558] Laranjo J., Bernier A., Meins E., Carlson S. M. (2014). The roles of maternal mind-mindedness and infant security of attachment in predicting preschoolers’ understanding of visual perspective taking and false belief. Journal of Experimental Child Psychology.

[B26-behavsci-15-01558] Li Z., Li B., Tsou Y. T., Wang L., Liang W., Rieffe C. (2024). A longitudinal study on moral emotions and psychosocial functioning among preschool children with and without hearing loss. Development and Psychopathology.

[B27-behavsci-15-01558] Lind-Combs H. C., Holt R. F. (2022). Associations between parent mental state language and child inhibitory control in children who are deaf or hard of hearing. Journal of Speech, Language, and Hearing Research.

[B28-behavsci-15-01558] MacWhinney B. (2000). The CHILDES project: Tools for analyzing talk.

[B29-behavsci-15-01558] Marzi G., Balzano M., Marchiori D. (2024). K-alpha Calculator–Krippendorff’s alpha calculator: A user-friendly tool for computing Krippendorff’s Alpha inter-rater reliability coefficient. MethodsX.

[B30-behavsci-15-01558] Moeller M. P., Schick B. (2006). Relations Between Maternal Input and Theory of Mind Understanding in Deaf Children. Child Development.

[B31-behavsci-15-01558] Morgan G., Meristo M., Mann W., Hjelmquist E., Surian L., Siegal M. (2014). Mental state language and quality of conversational experience in deaf and hearing children. Cognitive Development.

[B32-behavsci-15-01558] Netten A. P., Rieffe C., Soede W., Dirks E., Korver A. M. H., Konings S., Briaire J. J., Oudesluys-Murphy A. M., Dekker F. W., Frijns J. H. M. (2017). Can you hear what I think? Theory of mind in young children with moderate hearing loss. Ear & Hearing.

[B33-behavsci-15-01558] Pluta A., Krysztofiak M., Zgoda M., Wysocka J., Golec K., Gajos K., Dołyk T., Wolak T., Haman M. (2023). Theory of mind and parental mental-state talk in children with CIs. The Journal of Deaf Studies and Deaf Education.

[B34-behavsci-15-01558] Pluta A., Krysztofiak M., Zgoda M., Wysocka J., Golec K., Wójcik J., Włodarczyk E., Haman M. (2021). False belief understanding in deaf children with cochlear implants. The Journal of Deaf Studies and Deaf Education.

[B35-behavsci-15-01558] Roby E., Scott R. M. (2022). Exploring the impact of parental education, ethnicity and context on parent and child mental-state language. Cognitive Development.

[B36-behavsci-15-01558] Ruffman T., Slade L., Crowe E. (2002). The relation between children’s and mothers’ mental state language and theory-of-mind understanding. Child Development.

[B37-behavsci-15-01558] Schapira R., Aram D. (2020). Shared book reading at home and preschoolers’ socio-emotional competence. Early Education and Development.

[B38-behavsci-15-01558] Schick B., De Villiers P., De Villiers J., Hoffmeister R. (2007). Language and theory of mind: A study of deaf children. Child Development.

[B39-behavsci-15-01558] Soderstrom M., Wittebolle K. (2013). When do caregivers talk? The influences of activity and time of day on caregiver speech and child vocalizations in two childcare environments. PLoS ONE.

[B40-behavsci-15-01558] Stevenson J., Kreppner J., Pimperton H., Worsfold S., Kennedy C. (2015). Emotional and behavioural difficulties in children and adolescents with hearing impairment: A systematic review and meta-analysis. European Child and Adolescent Psychiatry.

[B41-behavsci-15-01558] Tamis-LeMonda C. S., Kuchirko Y. A., Escobar K., Bornstein M. H. (2019). Language and play in parent–child interactions. Handbook of parenting.

[B42-behavsci-15-01558] Taumoepeau M., Ruffman T. (2006). Mother and infant talk about mental states relates to desire language and emotion understanding. Child Development.

[B43-behavsci-15-01558] Tompkins V., Benigno J. P., Kiger Lee B., Wright B. M. (2018). The relation between parents’ mental state talk and children’s social understanding: A meta-analysis. Social Development.

[B44-behavsci-15-01558] Tompkins V., Montgomery D. E., Blosser M. K. (2022). Mother-child talk about mental states: The what, who, and how of conversations about the mind. Social Development.

[B45-behavsci-15-01558] van der Zee R. B., Dirks E. (2022). Diversity of child and family characteristics of children with hearing loss in family-centered early intervention in The Netherlands. Journal of Clinical Medicine.

[B46-behavsci-15-01558] Vygotsky L. S. (1978). Mind in society: The development of higher psychological processes.

[B47-behavsci-15-01558] Walker E. A., Ambrose S. E., Oleson J., Moeller M. P. (2017). False belief development in children who are hard of hearing compared with peers with normal hearing. Journal of Speech, Language, and Hearing Research.

[B48-behavsci-15-01558] Wellman H. M. (2018). Theory of mind: The state of the art. European Journal of Developmental Psychology.

[B50-behavsci-15-01558] Wiefferink C. H., Rieffe C., Ketelaar L., De Raeve L., Frijns J. H. M. (2013). Emotion understanding in deaf children with a cochlear implant. The Journal of Deaf Studies and Deaf Education.

[B49-behavsci-15-01558] Wiefferink C. H., Rieffe C., Ketelaar L., Frijns J. H. M. (2012). Predicting social functioning in children with a cochlear implant and in normal-hearing children: The role of emotion regulation. International Journal of Pediatric Otorhinolaryngology.

[B51-behavsci-15-01558] Ziv M., Smadja M. L., Aram D. (2013). Mothers’ mental-state discourse with preschoolers during storybook reading and wordless storybook telling. Early Childhood Research Quarterly.

